# Lead and Other Trace Element Levels in Brains of Croatian Large Terrestrial Carnivores: Influence of Biological and Ecological Factors

**DOI:** 10.3390/toxics11010004

**Published:** 2022-12-20

**Authors:** Maja Lazarus, Ankica Sekovanić, Slaven Reljić, Josip Kusak, Maja Ferenčaković, Magda Sindičić, Tomislav Gomerčić, Đuro Huber

**Affiliations:** 1 Institute for Medical Research and Occupational Health, 10000 Zagreb, Croatia; 2 Faculty of Veterinary Medicine, University of Zagreb, 10000 Zagreb, Croatia; 3 Faculty of Agriculture, University of Zagreb, 10000 Zagreb, Croatia; 4 Institute of Nature Conservation, Polish Academy of Sciences, 31-343 Krakow, Poland

**Keywords:** inorganic pollutants, metal(loid)s, brown bear (*Ursus arctos*), grey wolf (*Canis lupus*), Eurasian lynx (*Lynx lynx*), golden jackal (*Canis aureus*), apex predator, liver

## Abstract

Trace element pollution can adversely affect the brains of individuals and thus impact the entire population of apex predators, such as large European carnivores. We assessed exposure to prominent neurotoxicants As, Cd, Hg and Pb by measuring their brain stem levels in brown bears (*n* = 114), grey wolves (*n* = 8), Eurasian lynx (*n* = 3), and golden jackals (*n* = 2) sampled in 2015–2022 in Croatia. The highest of the non-essential elements was the Pb level in the bearsʼ brains (median, Q1–Q3; 11.1, 7.13–24.1 μg/kg wet mass), with 4% of animals, all subadults, exceeding the established normal bovine levels (100 μg/kg wet mass). Species-specific differences were noted for Ca, Cd, Cu, Fe, Pb and Se brain levels. Female brown bears had higher As brain levels than males. Cubs and yearlings had lower brain Cd, but higher Zn, while subadults had higher Cu than adult bears. Hepatic As, Cd, Cu and Hg levels were shown to be a moderate proxy for estimating brain levels in bears (r_S_ = 0.30–0.69). Multiple associations of As, Cd, Hg and Pb with essential elements pointed to a possible interaction and disturbance of brain Ca, Cu, Fe, Se and Zn homeostasis. Non-essential element levels in the brains of four studied species were lower than reported earlier for terrestrial meso-carnivores and humans. The age and sex of animals were highlighted as essential factors in interpreting brain element levels in ecotoxicological studies of large carnivores.

## 1. Introduction

Neurotoxicological data originating from wildlife exposed to multiple pollutants and residing close to humans or sharing the same trophic level and longevity were emphasized as a good addition to otherwise ethically challenging human studies due to their relevance for human health [1,2]. Environmental trace element pollutants such as arsenic (As), cadmium (Cd), methylmercury (MeHg) and lead (Pb) adversely affect multiple body systems, among which the brain and central nervous system are the most sensitive, as shown in human, experimental animal and wildlife studies [3,4,5]. Environmental exposure to As, Cd, MeHg and Pb could occur simultaneously [6], and toxicity is characterized partly by confounding factors, such as age, sex, nutritional status or status of essential elements related thereto [1]. Non-essential elements are reported to compete with essential elements such as Ca, Cu, Fe or Zn for their transporters when crossing cellular membranes, disrupting their homeostasis and the normal structure and function of brain components [7]. Arsenic can induce oxidative stress in brains, interact with cytoskeletal proteins in neurons, deplete sulfhydryl groups, substitute phosphate in ATP and alter key gene expression [3,7,8,9]. Cadmium interferes with Ca metabolism, inhibits thiol-rich enzymes, depletes antioxidative defense, and decreases levels of serotonin and acetylcholine [5,10]. The toxic mechanisms of MeHg include enhancement of oxidative stress, perturbation of Ca, Cu, Se and Zn homeostasis, formation of complexes with thiol-rich proteins and peptides, changes in glutamate and gamma-aminobutyric acid (GABA) signaling, as well as cellular signaling and gene expression. Inorganic Hg in the brain is believed to be a consequence of MeHg demethylation and to have lower neurotoxicity potential than MeHg, as it is sequestered by metallothioneins [11,12]. Lead also induces oxidative stress, substitutes Zn and Ca in enzymes and proteins, and alters glutamatergic, dopaminergic, and cholinergic neurotransmitter systems [13,14,15]. No level of human exposure to Pb is considered safe [16]. The developmental aspect of neurotoxicity caused by the mentioned non-essential elements attracts special attention. The brain is limited in its ability to recover and compensate for toxic effects; thus, an initiated impairment can be life-long [7,17] while affecting individuals and the population [1,18]. Particularly vulnerable windows of susceptibility (the prenatal and early postnatal periods) are characterized by subtle and complex changes in brains involving cell multiplication, differentiation, migration and synapse formation [17,19], underdeveloped blood-brain barriers (BBB), and sequestration and excretion mechanisms, all of which exacerbate the adverse effects in juvenile and adult stages with even low-level exposures [8,9,10,14,20].

Lead and Hg are prominent neurotoxicants recognized as threats to terrestrial wildlife with environmental exposure [21,22]. In free-living raptors and other bird species Pb, Cd and Hg were associated with effects on brain receptors and enzymes (N-methyl-D-aspartic acid, NMDA; glutamic acid decarboxylase, GAD; and glutamine synthetase, GS [23,24]), and exploration behavior (just Pb [25]) in animals with blood or liver levels above thresholds for Pb toxicosis [26]. However, changes in behavioral patterns were also noted in birds with background Pb blood levels [27,28]. Published studies in mammals focused almost exclusively on Hg-related neurotoxicity in (semi)aquatic species such as the mink (*Mustela vison*), river otter (*Lontra canadensis*), pinnipeds, cetaceans or polar bears (*Ursus maritimus*) in significant numbers of individuals [1,2,29,30,31,32,33]. Mercury levels lower than thresholds for neurotoxicity (established in laboratory animal and wildlife studies [33]) were associated with changes in various neurochemical receptors and enzymes (NMDA; muscarinic cholinergic, mACh; GABA; dopamine, D2; monoaminoxidase; and cholinesterase) in the wild and captive mink, river otter and polar bear (reviewed in [34]). Mercury has rarely been investigated in terrestrial mammalian brains and has been investigated in few animals: 29 Egyptian mongooses (*Herpestes ichneumon*) from Portugal [35], 6 red foxes (*Vulpes vulpes*) and 14 raccoon dogs (*Nyctereutes procyonoides*) from Russia [36], and 13 raccoons (*Procyon lotor*) from Poland [37]. Brain Pb and Cd, in addition to the aforementioned Hg, were included in a Polish report on 6 badgers (*Meles meles*), 9 pine martens (*Martes martes*) and beech martens (*Martes foina*), 14 red foxes, 17 raccoons and 12–15 raccoon dogs [38], all meso-carnivores. However, to the best of our knowledge, these elements (Cd, Hg, and Pb) were not measured in the brain of any large European carnivore. Large carnivores are at the top of the trophic pyramid and therefore accumulate more non-essential elements than their prey [39], sometimes approaching toxicologically relevant levels of Cd and/or Pb in the liver and kidneys, as found in 1–5% of Croatian brown bears (*Ursus arctos*, [40]). Furthermore, Pb serum levels in this population were associated with altered antioxidative enzyme superoxide-dismutase activity [41]. Lead levels and changes in microstructural properties of gray matter were reported in Japanese humans [42], with Pb hair content similar to those in Croatian brown bears (mean 397 vs. 401 μg/kg, respectively [41,42]). On those grounds, we hypothesized that with chronic exposure, some non-essential element burden may reach the brain and adversely affect some bears and other sympatric large and medium predators. Our concern especially relates to brown bear cubs, as they are in a susceptible life stage with reported renal Pb levels that match their mothers, which may have detrimental impact on their developing brains, taking into account prolonged exposure to Pb, Cd and Hg through suckling [43]. Except for the already mentioned age of carnivores, sex, season and body condition as determinants of an animal’s health are important confounding factors that have to be taken into consideration while interpreting tissue element levels [40,41,44,45,46]. It should be stressed that Croatian bears and other predators are concomitantly exposed to trace elements and organic pollutants with toxic potential [40,47,48]. The health of those protected species (brown bear, grey wolf (*Canis lupus*) and Eurasian lynx (*Lynx lynx*); EU Habitats Directive, Bern Convention) is of both national and European interest [49], Croatia being one of the rare European countries with all three large carnivore species [50]. The importance of large carnivores as sentinel species was argued earlier [1,2], and the wide distribution of the golden jackal (*Canis aureus*), with persistent expansion to western European countries, availability of samples due to categorization as a game species, its opportunistic predation and omnivorous diet, and its ability to live close to humans [51], also make it a valuable monitoring species. However, how accurately element levels in the jackalsʼ tissues reflect environmental fluctuations still has to be confirmed.

Our aims were to: (a) discuss non-essential trace element brain levels found in one medium-sized and three large carnivores from Croatia and compare them with the available threshold levels for neurotoxicity; (b) assess the influence of age, sex, body condition index (BCI) and season as relevant factors modifying the trace element levels in brown bears; (c) explore the possibility of estimating brain trace element levels from the more easily available hepatic tissues of bears and wolves; and (d) assess the interactions of non-essential elements with levels of homeostatically regulated elements in the brains of bears and wolves.

## 2. Materials and Methods

### 2.1. Animal Sampling

Lower portions of the brain stem (*medulla oblongata* [52]) were obtained from 114 brown bears (period 2015–2018), 8 grey wolves (2016–2017), 3 Eurasian lynx (2017–2022) and 2 golden jackals (2017) from Croatia (app. 43.5°–46° N, 14.5°–16.5° E). As a number of previous studies showed generally similar Hg levels across different brain regions (reviewed in [2] for polar bears and (semi)aquatic mammals), we chose to sample the brain stem as the easiest accessible brain region in large carnivores. In addition, paired liver tissues were collected from 49 bears and 8 wolves and provided, along with brain tissues, by the Faculty of Veterinary Medicine, University of Zagreb and Croatian Ministry of Agriculture. Brain and liver tissues were sampled from bears hunted according to the Brown Bear Management Plan [53] and/or road and railway casualties (bears, wolves, lynx and jackals) within ongoing research and conservation projects. None of the animals were killed for the purpose of this study. Body mass, body length, sex, date of death and location were recorded on site. The first premolar tooth was extracted from every bear to determine the age using counts of cementum annuli [54], while the age of wolves [55], lynx [56] and jackals were estimated on the basis of body size, tooth wear and the date of death. Bears were considered adults at the age of 4 years and older [43,57], subadults at the age of 2 years and less than 4 years, yearlings at the age of 1 year, while cubs were less than a year old. Wolves were categorized as adults at age 2 years and older [58,59]. Of the collected lynx individuals for this study, one was a cub (male), one a yearling (male) and one an adult (female), while both jackals were adults (male and female). Samples were stored in plastic tubes at −20 °C until further analyses.

### 2.2. Element Analyses

Brain and liver tissues were acid-digested in quartz vessels to a colorless liquid in an UltraCLAVE IV (Milestone, Sorisole, Italy) microwave digestion system and diluted, and then elements (As, Ca, Cd, Cu, Fe, total Hg, Pb, Se and Zn) were quantified by means of ICP-MS (Agilent 7500cx, Tokyo, Japan) according to a previously published method [60]. Purified nitric acid (65%, Merck, Darmstadt, Germany; duoPUR, Milestone, Sorisole, Italy) and ultrapure water (GenPure system, TKA, Niederelbert, Germany) were used in all steps. Certified reference materials (CRM) BCR-185R Bovine liver and BCR-186 Pig kidney (Institute for Reference Materials and Measurements, Geel, Belgium) were included in each digestion series and measured in duplicate with samples for method quality control. Recovery rates of elements in CRMs ranged 92–110%. All results are expressed on a wet-mass basis. Brain results were converted to a dry-mass basis (to enable literature comparisons) with a conversion factor of 3.7 based on the average water percentage of different brain regions and of the brain stem (73%) reported for polar bears [29,31]. Brain water content was shown to vary largely between species [1] and even between brain regions (13 regions [61]), so we applied data for the species closest to the brown bear, which is the polar bear.

### 2.3. Statistical Procedure

Data below the method detection limit (MDL) were replaced with half of the value of the MDL during statistical analysis. For the purpose of calculating Se/Hg molar ratios, Se and Hg levels were first divided by the molar weight (78.96 and 200.59 g/mol, respectively). As nearly all variables showed non-normal distribution (except hepatic Se), raw data were presented with median, interquartile range (IQR, 25th and 75th percentile, Q1 and Q3), minimum and maximum values. Biological (age, sex, and BCI) and sampling factors (season) were tested as factors influencing variations in only bear and wolf element levels. Body condition indexes of bears were calculated according to an equation set for North American brown bears (grizzly) and American black bears (*Ursus americanus*): BCI = (lnM − 3.21 × lnL + 11.64)/(0.29 − 0.017 × lnL) [46]. Bears were categorized into spring (March–August) or fall (September–February) groups according to the dates when they were sampled [40]. First, each element was tested separately for the influence of that respective factor using a Student’s *t*-test or an ANOVA with Tukey HSD test if the data from brain or liver tissue were normally distributed (Shapiro–Wilk’s test) and the difference in variances between groups was not significant (Levene’s test), while the Mann–Whitney *U* test or Kruskal–Wallis test was used otherwise. Associations between elements in two tissues, different elements within the same tissue, or elements and age or BCI were tested using Spearman’s correlation coefficient. Multivariate linear regression analyses were finally performed to identify significant factors adding to variations in brown bear element levels, while sample sizes of wolf data were insufficient for such analysis. Variable transformation (log_10_ or square root), residual analyses and a check on Cook’s distance were carried out to confirm the fulfilment of conditions for regression analyses. Statistics and visualization were performed using TIBCO Statistica^®^ software, version 14.0.0.15 (TIBCO Software Inc., Palo Alto, CA, USA) and SAS 9.4 software (SAS Institute Inc., Cary, NC, USA).

## 3. Results

Sample sizes were large enough to enable age and sex group comparisons within the biometric data (Table 1) only for the brown bear. Subadult and adult brown bears differed between sexes in body mass and length, which is in line with well-established sexual dimorphism in this species triggered by the onset of maturity [62].

### 3.1. Between-Species Differences in Trace Elements

Due to the restrictions in the number of collected specimens, we were able to statistically compare element levels only between bears and wolves (Table 2), while the results gained from lynx and jackals are listed in Table S1, together with brain element data from other large and medium-sized terrestrial mammalian carnivores and humans for comparison. Element levels measured in brain and liver tissue showed distinct variation between bears and wolves (Table 2). Cadmium, Cu and Pb were higher in bears’ brain and liver tissue than in wolves’, while Fe and Se were higher in the brain and liver tissue of wolves. In addition, bears had higher hepatic As, Hg and Zn levels compared to wolves.

### 3.2. Influence of Age, Sex, BCI, and Season on Trace Elements in the Brains of Brown Bears

**Figure 1:**
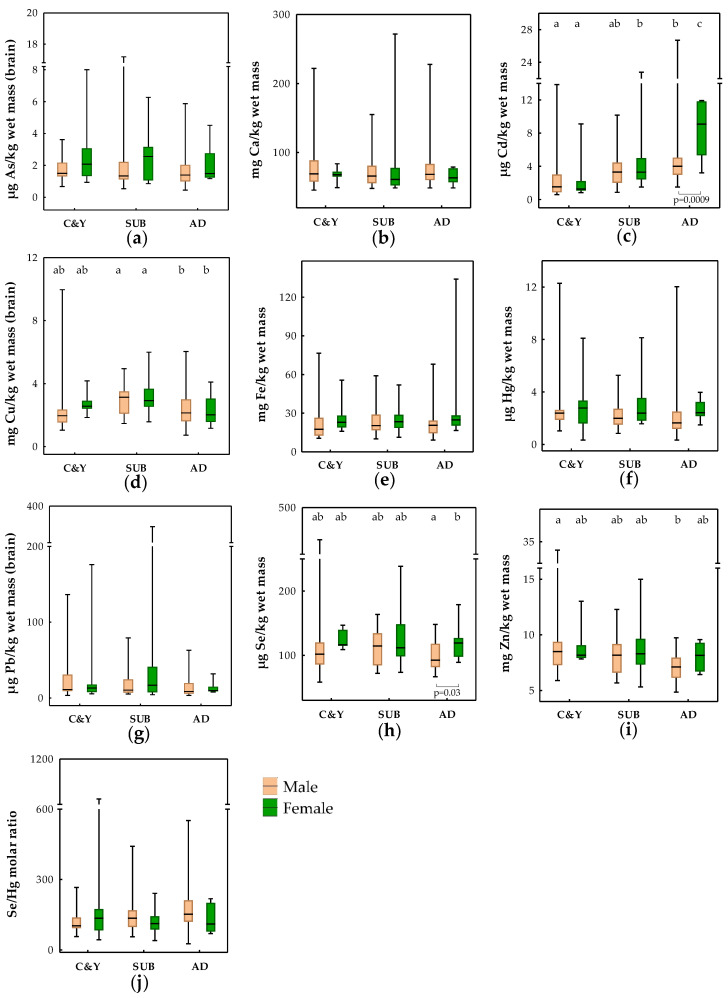
Age group and sex differences in the brain levels of (**a**) As; (**b**) Ca; (**c**) Cd; (**d**) Cu; (**e**) Fe; (**f**) Hg; (**g**) Pb; (**h**) Se; (**i**) Zn; and (**j**) Se/Hg molar ratios in brown bears from Croatia. Boxes denote interquartile range (IQR), the line in the box is the median, and whiskers denote minimum and maximum values. Significant differences between age groups within the same sex are marked with different letters above the boxes (a,b,c), while for sex differences within one age group, the level of significance is additionally noted below the boxes. C&Y—cubs and yearlings; SUB—subadults; AD—adults.

Element levels measured in the brains of brown bears are presented in Figure 1 and categorized according to age group and sex. The influence of the mentioned biological factors (age and sex) together with BCI and season of sampling on element levels was explored using linear regression analyses (Table S2). Lead results were not included in Table S2 as conditions for regression analyses were not fulfilled for this element. Sex was shown to have an important effect on As variation in the brains of brown bears (noted also in univariate testing shown in Figure 1; lower levels in males), while age group was highlighted as a factor influencing Cd, Cu and Zn brain levels (Table S2). Cubs and yearlings had lower Cd, but higher Zn, while subadults had higher Cu than adult bears.

### 3.3. Association of Trace Elements between the Brain and Liver

**Figure 2:**
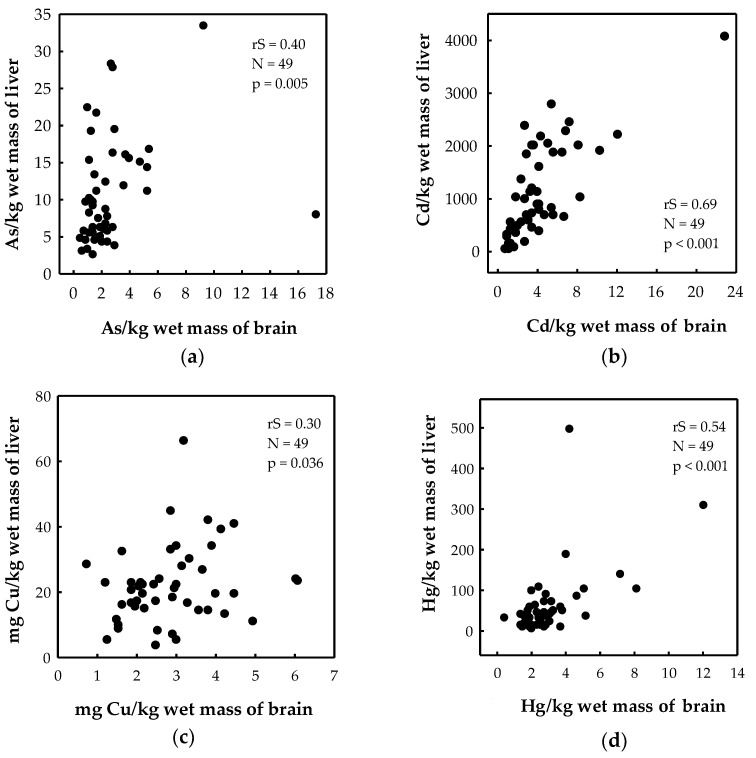
Correlation of (**a**) As; (**b**) Cd; (**c**) Cu; and (**d**) Hg levels between the brain and the liver of brown bears from Croatia. r_S_—Spearman correlation coefficient; N—number of individuals; *p*—level of significance.

Livers contained higher levels of elements compared to brains in both bears and wolves (Table 2). Liver-to-brain ratios in bears were 6 for As, 272 for Cd, 18 for Hg and 53 for Pb. The same ratios in wolves were 3 for As, 71 for Cd, 4 for Hg and 16 for Pb. A significant correlation between the brain and liver was noted for As, Cd, Cu, and Hg levels (Figure 2) and Se/Hg molar ratios (r_S_ = 0.50, *p* < 0.001, *n* = 49) in brown bears. No significant element correlations were found between the two tissues in wolves, although this result should be taken with caution because of the low number of sampled individuals (*n* = 8).

### 3.4. Association between Non-Essential and Essential Trace Elements in the Brain and Liver

**Figure 3:**
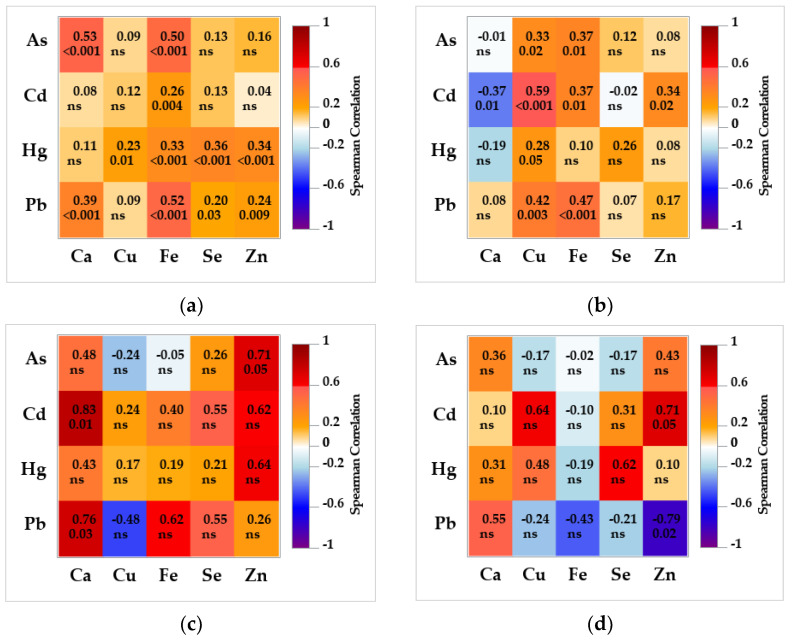
Heatmap of Spearman correlation coefficients between toxic and essential elements in (**a**) brown bear brain (*n* = 114); (**b**) brown bear liver (*n* = 49); (**c**) grey wolf brain (*n* = 8); and (**d**) grey wolf liver (*n* = 8) from Croatia. The level of significance is denoted below the coefficient; otherwise, non-significant (ns) symbol is stated.

A heatmap of Spearman rank correlation coefficients (Figure 3) between non-essential and essential elements revealed more significant associations in the tissue of bears than wolves, but of lower degree (bears: 0.20 < r_S_ ≤ 0.59; wolves: 0.71 < r_S_ ≤ 0.83). In the bear brains, Fe was significantly correlated with all four non-essential elements; Cu, Se and Zn were correlated with Hg; while Ca, Se and Zn were also correlated with Pb. Calcium was also significantly correlated with As. In the brains of eight wolves, only As was highly correlated with Zn, and Ca with Cd and Pb. In the bear liver, Cu and Fe were significantly correlated with all non-essential elements (except Fe with Hg), and Ca and Zn with Cd. Zinc was highly correlated with Pb and Cd in the liver of wolves.

## 4. Discussion

This is the first report on brain essential and non-essential elements in the brown bear, grey wolf, Eurasian lynx, golden jackal, and large terrestrial carnivores in general, except for studies on polar bears linked to the marine environment [1,2,29,31,32]. To assess the risk of Hg or Pb-related effects on the brain, we compared measured levels with established threshold values for neurotoxicity in mammals (Hg) or values set as normal for domestic animals (Pb), due to the lack of more relevant data for brains in large predators. 

The highest Hg levels in bears (12.3 μg/kg wet mass), wolves (6.25 μg/kg wet mass), lynx (19.0 μg/kg wet mass) and jackals (73.9 μg/kg wet mass) studied here were below threshold values for neurobehavioral effects established in laboratory animals (100 μg/kg wet mass) or the lowest observed adverse-effect level (LOAEL; 400 μg/kg wet mass) for neurochemical changes set in captive mink (reviewed in [33]). The range of Hg brain values in our bears was at least one order of magnitude lower than polar bears’ levels associated with a reduction in NMDA [2,29] and neurochemical enzyme activity (monoamine oxidase [32]). Bald eagles (*Haliaeetus leucocephalus*) with Hg-associated changes in GS, GAD and NMDA had brain levels more than two orders of magnitude higher than our carnivores [24]. Thus, we would not expect, a priori, Hg-related adverse effects in the brains of Croatian carnivores.

Brain Pb levels considered normal for cattle (100 μg/kg wet mass [63]) were exceeded in five young bear individuals (a male cub, a male and female yearling and two female subadults) out of total of 74 young bears. Although bear Pb levels were below what is considered high-Pb content for cattle (>500 μg/kg wet mass [63]), because the high neurotoxic potential of Pb in young animals [7,14,17] might affect the developing brain in bears, we cannot exclude adverse health effects. 

Scarcely reported Pb brain levels from American bald eagles associated with changes in brain GS and GAD activity [23] were at least 40 times higher compared to our carnivores’ brains, while liver tissues showed a smaller distinction (eagles: 4160 μg/kg wet mass vs. 590 and 120 μg/kg wet mass in bears and wolves, respectively; recalculated from dry mass on the basis of 68% H_2_O). Less consistent with differences between American and Croatian brain levels, hepatic Pb in European raptors ranged (mean: 570 μg/kg wet mass, min-max: 11–5760 μg/kg wet mass [64]; recalculated from dry mass on the basis of 68% H_2_O) similar to bears and higher than in wolves from this study. Blood Pb levels in environmentally exposed raptors and songbirds exhibiting behavioral changes (100 μg/L, [28]; 785 and 894 μg/kg wet mass [25]) and in chronically exposed mammalian species with confirmed neurotoxic effects (70–200 μg/L [65]) was in the range found in our brown bear population (5–168 μg/L [41]). A study in developing young adult humans with hair Pb levels similar to those in Croatian bears (0.397 vs. 0.401 mg/kg [41]) found weak Pb-related effects on the excitability and microstructural properties of the brain [42]. Future effect studies on bear brains could confirm our conclusions on the scale of environmental, especially Pb-related, effects.

### 4.1. Between-Species Differences in Trace Elements

Lead was the non-essential element with the highest concentrations in the brains of bears and wolves, as reported in meso-carnivores [38]. These authors characterized Pb, Cd and Hg levels in Polish meso-carnivores as background, although Pb was two to five times higher than in our four studied species. Croatian carnivores had lower-to-comparable Cd brain levels with Polish ones [38].

Mercury brain levels in Croatian carnivores were comparable to the Polish red fox [38] and American raccoon [66], but three to ten times lower than those found in other terrestrial predators (Table S1).

Of Croatian carnivores, brown bears had the lowest Ca level, but the highest Cd and Pb levels. The exposure sources resulting in high Hg levels found in three lynx and one jackal brain are unknown, and the low numbers mean that firm conclusions about species differences cannot be drawn. Furthermore, a study on muscle, liver and kidney tissue variations between the same species did not confirm similar Hg peaks in the lynx and jackal [40]. Species-specific soil and/or sediment intake during feeding could add significantly to metal exposure (soil being 9% of the diet in raccoons and 3% of the diet in red fox [67]). The higher average age of animals per studied species (3.8 years for bears and 1.9 years for wolves) could increase total exposure times and consequently raise non-essential element tissue levels. While diet (element content in food, the chemical form of elements or macronutrient composition of food) was often held largely responsible for differences in tissue element levels between species [38,40], other physiological specificities (absorption, distribution, metabolism, and excretion of elements) of the respective species were also highlighted as important contributors [1]. Some authors have proposed that different rates of MeHg demethylation to inorganic Hg, higher intake or excretion of Hg caused by the absence or presence of different enzymes and transporters, BBB components, or available Se or thiol-containing proteins for sequestration or else, could add to interspecies variations in Hg brain levels [2,31]. Data on essential elements in the brains of terrestrial carnivores are scarce. Available essential element data for polar bears, raccoons and humans (Table S1) showed no significant discrepancies from the carnivores studied here (except for higher Se, followed by higher Hg levels, an association discussed later).

### 4.2. Influence of Age, Sex, BCI, and Season on Trace Elements in Brains of Brown Bears

Age influenced Cd, Cu and Zn levels in the brains of Croatian bears (Table S2), which is consistent with previous findings for muscle, liver and kidneys of the same bear population and Croatian wolves [40]. Because of the long half-life and protein sequestration and deposition primarily in target organs (kidney and liver), Cd accumulates in the organs with age until it plateaus in old age [40,68,69]. We observed a certain level of Cd accumulation with age in the brains of bears, which implies some degree of effective transport and deposition in the brain, even in adults. Also, an additional entrance for airborne Cd through the nose and olfactory bulb to the central and peripheral neurons might also occur in bears, as reported for humans as an alternative Cd pathway to the brain [70]. More precisely, mature BBB is known to considerably block the entrance of Cd from the blood to the brain, probably due to its binding to the low-molecular-weight protein, metallothionein (MT), in the blood. On the other hand, free Cd was reported to decrease the permeability of both mature and immature BBB in rats (reviewed by [5,71]). The permeability of the BBB as a function of age is lowest in immature and aged mammals, and higher in adults [5]. Generally serving as a Zn and Cu transporter, MT also sequesters non-essential metals, such as Cd, which greatly modulates its toxic effect [69]. All three MT isoforms are expressed in human and rodent brains, and both expression and protein levels increase with age, as does Cd, followed by declines in old age [72]. As Cd binds MT by displacing Zn from the protein, thereby enhancing MT mRNA expression, and Cd competes with Zn and Cu for the same membrane transporters (e.g., divalent metal transporter 1, DMT1; zinc-regulated, iron-regulated transporter, ZIP [73,74]), we can assume that the age association, with otherwise homeostatically regulated essential Zn and Cu levels, is due more to the interplay of accumulated Cd and MT with the essential elements mentioned. Our observation that the lowest Cd in cub and yearling bears occurred towards the highest Zn in the same age group (Table S2) might support an antagonistic relationship at the transporter level. Cd in the brains of cubs and yearlings was derived from their mother’s available Cd burden, transferred in limited amounts through the placenta and milk and through the crude diet introduced gradually after the fourth postnatal month [43,75,76]. The lack of effective studies on wild terrestrial mammals prevents us from making any conclusions on the possible effect of Cd levels found in the bearsʼ brains, but the evidence of effects on the dopaminergic biomarkers related to blood Cd levels below 0.5 μg/L in European children [70] should prompt our future study efforts in that direction, as blood Cd in the Croatian population of bears ranged even higher (<0.247–1.21 μg/L [41]). Unlike Cd, brain levels of the other developmental neurotoxicants, known for their even more effective prenatal and postnatal transfer compared to Cd, i.e., As, Hg and Pb, did not differ between the youngest and adult bear classes, which raises concerns about the adverse effects in cubs and yearlings, considering the confirmed presence and toxic potency [8,10,14], and the known vulnerability of young mammals [17]. The importance of Pb and Hg mother–cub transfer in Croatian brown bears was emphasized in a study focusing on renal tissue [43]. Opposite to Croatian bears, age-related decreases of Cd were reported in 17 wild raccoon brains [38], while Hg data from terrestrial wildlife, mainly the polar bear, were inconclusive [2,29,31,32]. Juvenile and adult Egyptian mongooses from Portugal did not differ in Hg brain levels (*n* = 20, [35]), while Lanocha et al. [37] found higher Hg in the brains of eight adult than five immature raccoons from NW Poland.

Numerous structural and functional differences driven by genetic, hormonal and environmental factors in humans cause differences in the toxicokinetics of elements between the two sexes [77,78,79]. Epidemiological studies reviewed by Tyler and Allan [80] suggested a more pronounced effect of low chronic As exposure on females than males, while Thakur et al. [9] stated As-related toxicity is more common in males. Regression modelling in our study showed female bears had higher As in brain samples than males (Table S2), as previously shown for As in muscle and liver tissue, Cd in liver and kidney tissue, and Hg in liver tissue [40]. This may indicate a similar and comprehensive influence of the aforementioned sex-factor on the toxicokinetics of elements in different tissues of bears. Mercury showed no sex-related variation in brain samples of polar bears (*n* = 24–107) [2,31,32], raccoon dogs [36] and Egyptian mongooses [35], while Kalisinska et al. [38] omitted sex as a factor contributing to element levels in meso-carnivores.

### 4.3. Association of Trace Elements between the Brain and Liver

Our aim was to compare trace element levels in the brains and livers of bears and wolves with effective sample sizes, and to investigate whether brain element levels can be predicted from the element levels measured in the liver. More precisely, livers are easier to extract from many animals than brains (especially in big mammals, such as bears), they are the tissues of highest relevance for the metabolism, detoxification and excretion of the majority of non-essential elements, and they are the most often studied tissues for monitoring wildlife with established threshold values [34]. Although the liver is burdened with higher levels of non-essential elements compared to the brain [1,31,35,36,37], the latter is more vulnerable to adverse effects, which can be demonstrated years and decades after exposure [2,8,9,14]. Liver-to-brain ratios for all non-essential elements were higher in Croatian bears than in wolves, pointing at more efficient protection mechanisms against neurotoxicity in bears including both reduced sequestration to the brain and/or enhanced elimination from the brain, as proposed earlier in polar bears [29]. Calculated liver-to-brain ratios for Hg in both large carnivore species (4 and 18 for wolves and bears, respectively) were comparable to the ratio from Egyptian mongooses (2 [35]), red foxes (10) and raccoon dogs (17 [36]), raccoons (21 [37]) or humans (2–21; reviewed in [31]), but much lower than reported for polar bear (326 [31]). Low- (for As and Cu) to-moderate correlations (for Cd and Hg) between the 49 liver and brain levels of brown bears obtained in this study indicate the limitation in using hepatic element data as a proxy for levels in bear brains, and vice versa. The available literature shows stronger associations between liver and brain Hg levels in raccoons [37] and marine mammals [1] and their absence in polar bears [1] and raccoon dogs [36].

### 4.4. Associations between Non-Essential and Essential Trace Elements in the Brain and Liver

Associations between non-essential and essential trace elements found here in the brains and livers of bears and wolves originate from their well-established interaction (e.g., replacement, antagonism or indirect depletion) with the transporter or other cell structural elements described in humans and experimental animals [7,11,15,73] that were also confirmed in earlier wildlife studies [40,81,82,83]. Consequently, a varying degree of enhancement or decrease in As, Cd, Hg or Pb-related toxicity may occur in the organism. The mechanisms underlying the noted associations of As with Ca, Cu, Fe and Zn in the brains and livers of bears and wolves are not well understood and may well have origins in the As affinity to protein sulfhydryls containing the mentioned essential elements [9,84]. Arsenic was proven to interact with certain Zn finger motifs and to disrupt this essential protein [84,85]. Cadmium and Ca were highly correlated in wolf brains, which is consistent with the described mechanism of Cd-related neurotoxicity based on ion mimicry involving competition with Ca ions and impairment of Ca levels, an important secondary messenger in the brain [5]. Hepatic associations of Cd with Ca, Cu, Fe and Zn probably reflect the competitive nature of Cd ions towards the essential elements on their transporting proteins in the intestines, blood and liver [73,74,84]. Our correlation analysis found low associations of brain Hg with Ca (wolves), Cu, Fe, Se and Zn (bears). The interaction mechanism is reported to depend on the chemical form of Hg (inorganic vs. organic, MeHg). In contrast to the liver, the prevailing form of Hg in polar bear and raccoon brains is MeHg (83–100% MeHg of total Hg), and this percentage was shown to decrease with increasing age [29,31,86]. Assuming the similar chemical form of Hg in our brown bears as in the mentioned carnivores, and a high affinity for sulfhydryl groups, we can assume the present MeHg crossed the BBB conjugated to some thiol-rich protein acting as a mimic of the methionine [73]. Once in the brain, MeHg was shown to disrupt the homeostasis of the Ca ion through the disruption of the glutamate transport [12]. Interaction of Hg with Cu and Zn in our carnivores may be caused by displacement of Zn from the MT or redox-regulating, metal-responsive transcription factors after Hg binds to DNA [11]. The most studied interaction of Hg with an essential element is that with Se and Se-containing compounds resulting in Hg sequestration, especially in the liver, but also in the kidney and brain [11,34]. Previous wildlife studies reported concurrent accumulations of Hg and Se in the liver and kidney [47,87,88,89,90], but brain samples did not follow this trend due to the limited Hg levels which were greatly surpassed by Se levels in polar bears [29], as well as in our brown bears and wolves. The Se/Hg molar ratios in Croatian bear and wolf brains were similar (128 and 161, respectively) and more than 20 times higher than in polar bears’ brain stems (5.6 [29]). Molar ratios of unity or lower imply that the available Se levels are not enough to detoxify Hg presently, binding it to an inert complex that precipitates [91]. The median liver Se/Hg molar ratio in our bears was similar to the ratios reported previously for Croatian bears (26 vs. 34, respectively [47]), but five times lower than in the livers of Croatian wolves. Such results reflect lower Hg levels in wolf livers and higher Se levels compared to bears, meaning that wolves are at lower risk of Hg-related toxicity. We found low-to-moderate correlations of Pb with Ca and Fe in bear and wolf brains (only Pb/Ca in wolves), which may reflect divalent cation mimicry, which is, together with oxidative stress, described as the main mechanism of Pb-associated neurotoxicity [15]. Competition of Pb with Ca, less pronounced with Zn and Fe, disrupts the N-methyl-D-aspartate receptor (NMDAR) and NMDAR-mediated calcium signaling involved in learning, memory and synaptic plasticity [15,92]. Hepatic associations of Pb with Cu, Fe and Zn, seen in our two large carnivores, may result from competition for the same binding sites on intestinal metal ion transporters (e.g., DMT1), which issues enhanced Pb absorption in conditions of Fe or Zn deficiency [15,84,93].

## 5. Conclusions

Data on elements in the brains of large terrestrial carnivores are generally lacking. This report is the first to focus on this area and includes brown bear, grey wolf, Eurasian lynx and golden jackal brain samples. Among Croatian carnivores, the brown bear had the highest Cd and Pb brain levels, which were, together with Hg and As, globally lower than in polar bears and the few reported European meso-carnivores. Species was the prominent cause of variation in brain element levels, followed by age and sex. In addition, essential elements were confirmed as important for brain accumulation of non-essential elements, which might affect their homeostasis but also reduce the risk of neurotoxic effects (such as Se in the case of Hg). Liver and brain element levels were moderately correlated in bears and wolves. Hg-related adverse effects in the brains of Croatian bears and wolves are not expected but Pb-related adverse effects cannot be excluded, as Pb levels in the few young individuals exceeded the normal levels set for cattle. Cub and yearling bears, being in their most susceptible period for neurotoxicological effects, had As, Hg and Pb brain levels comparable to adults, which calls for future studies focusing on Pb effects in the young brain, to rule out possible adverse effects in the highest centile of the exposed population.

## Figures and Tables

**Table 1:** The biometric data (median (IQR: Q1, Q3); N) of brown bears and grey wolves from Croatia categorized by sex and age group ^1^.

	Brown Bear (*n* = 114)			Grey Wolf (*n* = 8)		
		Male	Female		Male	Female
Age (y)	C&Y	1.0 (0.5, 1.0); 14 ^A^	0.9 (1.0, 1.0); 9 ^A^	SUB	1; 1	0.9 (0.6, 1.3); 4
	SUB	2.0 (2.0, 3.0); 30 ^B^	3.0 (2.0, 3.0); 21 ^B^	AD	2.5 (2.4, 2.5); 2	5.5; 1
	AD	5.0 (4.0, 10.0); 30 ^C^	6.0 (5.0, 9.0); 10 ^C^			
Body mass (kg)	C&Y	54.8 (30.1, 75.0); 14 ^A^	67.4 (40.7, 81.0); 8 ^A^	SUB	20; 1	24.2 (20.6, 24.6); 4
	SUB	135 (82.0, 190); 29 ^aB^	92.0 (71.0, 100); 21 ^bA^	AD	36.2 (35.0, 37.4); 2	28.3; 1
	AD	210 (179, 248); 30 ^aC^	130 (110, 185); 10 ^bB^			
Body length (cm)	C&Y	125 (117, 133); 14 ^A^	136 (114, 149); 8 ^A^	SUB	114; 1	107 (97.5, 117); 4
	SUB	175 (162, 185); 30 ^aB^	149 (143, 158); 20 ^bB^	AD	123 (122, 124); 2	120; 1
	AD	187 (180, 200); 29 ^aC^	160 (154, 170); 9 ^bB^			
BCI	C&Y	−0.09 (−0.49, 1.13); 14	0.61 (−0.99, 0.78); 8			
	SUB	0.32 (−0.77, 0.92); 30	0.55 (−0.73, 1.27); 20			
	AD	0.68 (0.09, 1.19); 29	1.46 (0.76, 1.57); 9			

^1^ C&Y—cubs and yearlings; SUB—subadults; AD—adults. Significant differences between sexes within the same age group (^ab^) or age groups within the same sex (^ABC^) of brown bears were tested with a *t*-test, ANOVA with Tukey HSD test or Mann–Whitney *U* test and Kruskal–Wallis test, and marked with different superscript letters.

**Table 2:** Descriptive statistics (median (IQR: Q1, Q3) min-max) and interspecies differences in element levels expressed on a wet-mass basis of brain (*n* = 114) and liver tissue (*n* = 49) in brown bears and grey wolves (*n* = 8) from Croatia ^1^.

Element	MDL	Tissue	Brown Bear	Grey Wolf	*p*
As (μg/kg)	0.412	brain	1.52 (1.12, 2.56) 0.452–17.2	1.27 (0.88, 1.96) 0.556–2.63	NS
		liver	8.88 (5.55, 15.3) 2.60–33.7	3.49 (2.42, 5.18) 1.53–17.2	0.003
Cd (μg/kg)	0.155	brain	3.40 (2.22, 5.19) 0.582–26.7	0.535 (0.289, 0.890) 0.167–3.36	<0.001
		liver	926 (495, 1918) 81.9–4093	38.2 (22.1, 71.6) 16.5–137	<0.001
Ca (mg/kg)	0.667	brain	66.3 (56.5, 80.3) 45.6–271	124 (111, 154) 108–324	<0.001
		liver	40.7 (36.9, 52.2) 27.2–104	65 (51.1, 119) 28.0–302	0.02
Cu (mg/kg)	0.100	brain	2.59 (1.89, 3.31) 0.723–9.97	1.96 (1.22, 2.37) 1.09–2.81	0.02
		liver	21.3 (15.1, 27.4) 3.93–66.4	8.13 (4.09, 14.7) 3.14–33.0	0.004
Fe (mg/kg)	0.068	brain	21.2 (16.7, 28.3) 9.20–134	33.8 (26.3, 41.9) 23.3–92.9	0.003
		liver	131 (87.8, 190) 38.5–549	148 (110, 200) 99.8–222	NS
Hg (μg/kg)	0.668	brain	2.14 (1.57, 2.97) < MDL–12.3	2.04 (1.54, 2.89) 1.16–6.25	NS
		liver	39.5 (18.2, 64.7) 8.64–499	7.95 (6.25, 18.1) 4.84–26.1	<0.001
Pb (μg/kg)	0.454	brain	11.1 (7.13, 24.1) 3.45–372	7.68 (3.55, 11.8) 1.10–16.6	0.04
		liver	590 (310, 831) 143–4046	120 (72.3, 167) 51.3–258	<0.001
Se (μg/kg)	0.770	brain	112 (86.8, 130) 58.3–448	133 (117, 147) 107–150	0.03
		liver	406 (328, 449) 249–689	489 (424, 564) 370–630	0.01
Zn (mg/kg)	0.042	brain	7.89 (6.71, 9.04) 4.85–34.2	7.54 (6.95, 8.36) 5.82–11.1	NS
		liver	35.8 (32.4, 45.3) 21.1–93.3	26.1 (22.3, 29.4) 14.6–50.1	0.003
Se/Hg		brain	128 (94.9, 174) 26.2–1024	161 (119, 192) 58.9–328	NS
		liver	25.8 (13.1, 53.2) 2.96–123	144 (94.6, 197) 47.8–230	<0.001

^1^ MDL—method detection limit; *p*—statistical significance of differences between species within the same tissue tested with a *t*-test or Mann–Whitney *U* test; NS—non-significant; Se/Hg—ratio of molar concentrations calculated with molar weight of 78.96 and 200.59 g/mol for Se and Hg, respectively.

## Data Availability

The data presented in this study are available on request from the corresponding author. The data are not publicly available due to privacy issues.

## Supplementary Materials

The following supporting information can be downloaded at: https://www.mdpi.com/article/10.3390/toxics11010004/s1, Table S1: Recent literature data for selected elements (mean, median) in the brain of large and medium-sized terrestrial mammalian carnivores and humans expressed on a dry-mass basis. Table S2: Results of linear regression modelling of associations of trace elements in the brown bear brain with sex, age group, body condition index (BCI) and season.

## References

[1] López-Berenguer G., Peñalver J., Martínez-López E. (2020). A critical review about neurotoxic effects in marine mammals of mercury and other trace elements. Chemosphere.

[2] Desforges J.P., Mikkelsen B., Dam M., Rigét F., Sveegaard S., Sonne C., Dietz R., Basu N. (2021). Mercury and neurochemical biomarkers in multiple brain regions of five Arctic marine mammals. Neurotoxicology.

[3] Caito S., Aschner M., Lotti M., Bleecker M.L. (2015). Chapter Eleven—Neurotoxicity of metals. Occupational Neurology.

[4] Wu X., Cobbina S.J., Mao G., Xu H., Zhang Z., Yang L. (2016). A review of toxicity and mechanisms of individual and mixtures of heavy metals in the environment. Environ. Sci. Pollut. Res..

[5] Ruczaj A., Brzóska M.M. (2022). Environmental exposure of the general population to cadmium as a risk factor of the damage to the nervous system: A critical review of current data. J. Appl. Toxicol..

[6] Oliveira C.S., Nogara P.A., Ardisson-Araújo D.M.P., Aschner M., Rocha J.B.T., Dórea J.G., Aschner M., Costa L.G. (2018). Chapter Two—Neurodevelopmental effects of mercury. Advances in Neurotoxicology.

[7] Andrade V.M., Aschner M., Marreilha dos Santos A.P., Aschner M., Costa L. (2017). Neurotoxicity of metal mixtures. Advances in Neurobiology.

[8] Masjosthusmann S., Tigges J., Fritsche E., Koch K., Aschner M., Costa L.G. (2021). Chapter Two—Arsenic-mediated developmental neurotoxicity: Recent advances in understanding the adverse outcomes and underlying mechanisms. Neurotoxicity of Metals: Old Issues and New Developments. Advances in Neurotoxicology.

[9] Thakur M., Rachamalla M., Niyogi S., Datusalia A.K., Flora S.J.S. (2021). Molecular mechanism of arsenic-induced neurotoxicity including neuronal dysfunctions. Int. J. Mol. Sci..

[10] Gonçalves J.F., Dressler V.L., Assmann C.E., Morsch V.M.M., Schetinger M.R.C., Aschner M., Costa L.G. (2021). Chapter Three—Cadmium neurotoxicity: From its analytical aspects to neuronal impairment. Neurotoxicity of Metals: Old Issues and New Developments.

[11] Bjørklund G., Dadar M., Mutter J., Aaseth J. (2017). The toxicology of mercury: Current research and emerging trends. Environ. Res..

[12] Branco V., Aschner M., Carvalho C., Aschner M., Costa L.G. (2021). Chapter Seven—Neurotoxicity of mercury: An old issue with contemporary significance. Neurotoxicity of Metals: Old Issues and New Developments. Advances in Neurotoxicology.

[13] Cory-Slechta D.A. (1995). Relationships between lead-induced learning impairments and changes in dopaminergic, cholinergic, and glutamatergic neurotransmitter system functions. Annu. Rev. Pharmacol. Toxicol..

[14] Agency for Toxic Substances and Disease Registry (ATSDR) Toxicological Profile for Lead. Department of Health and Human Services, Public Health Service, Centers for Disease Control Atlanta, ATSDR, Atlanta, GA, USA, 2020. https://www.atsdr.cdc.gov/toxprofiles/tp13.pdf.

[15] Virgolini M.B., Aschner M., Aschner M., Costa L.G. (2021). Molecular mechanisms of lead neurotoxicity. Neurotoxicity of Metals: Old Issues and New Developments. Advances in Neurotoxicology.

[16] European Food Safety Authority (EFSA) (2010). Scientific opinion on lead in food. EFSA J..

[17] Grandjean P., Herz K.T. (2015). Trace elements as paradigms of developmental neurotoxicants: Lead, methylmercury and arsenic. J. Trace Elem. Med. Biol..

[18] Bertram M.G., Martin J.M., McCallum E.S., Alton L.A., Brand J.A., Brooks B.W., Cerveny D., Fick J., Ford A.T., Hellström G. (2022). Frontiers in quantifying wildlife behavioural responses to chemical pollution. Biol. Rev..

[19] National Research Council (U.S.) (1993). Pesticides in the Diets of Infants and Children. Committee on Pesticides in the Diets of Infants and Children.

[20] Landrigan P.J. (2005). Children as a vulnerable population. Int. J. Occup. Med. Environ. Health.

[21] Dietz R., Fort J., Sonne C., Albert C., Bustnes J.O., Christensen T.K., Ciesielski T.M., Danielsen J., Dastnai S., Eens M. (2021). A risk assessment of the effects of mercury on Baltic Sea, Greater North Sea and North Atlantic wildlife, fish and bivalves. Environ. Int..

[22] Chiverton L., Cromie R., Kock R. (2022). European mammal exposure to lead from ammunition and fishing weight sources. Heliyon.

[23] Nam D.H., Rutkiewicz J., Basu N. (2012). Multiple metals exposure and neurotoxic risk in bald eagles (*Haliaeetus leucocephalus*) from two Great Lakes states. Environ. Toxicol. Chem..

[24] Rutkiewicz J., Nam D.H., Cooley T., Neumann K., Padilla I.B., Route W., Strom S., Basu N. (2011). Mercury exposure and neurochemical impacts in bald eagles across several Great Lakes states. Ecotoxicology.

[25] Grunst A.S., Grunst M.L., Daem N., Pinxten R., Bervoets L., Eens M. (2019). An important personality trait varies with blood and plumage metal concentrations in a free-living songbird. Environ. Sci. Technol..

[26] Franson J.C., Pain D.J., Beyer W.N., Meador J.P. (2011). Lead in birds. Environmental Contaminants in Biota: Interpreting Tissue Concentrations.

[27] Ecke F., Singh N.J., Arnemo J.M., Bignert A., Helander B., Berglund Å.M.M., Borg H., Bröjer C., Holm K., Lanzone M. (2017). Sublethal lead exposure alters movement behavior in free-ranging golden eagles. Environ. Sci. Technol..

[28] McClelland S.C., Durães Ribeiro R., Mielke H.W., Finkelstein M.E., Gonzales C.R., Jones J.A., Komdeur J., Derryberry E., Saltzberg E.B., Karubian J. (2019). Sub-lethal exposure to lead is associated with heightened aggression in an urban songbird. Sci. Total Environ..

[29] Basu N., Scheuhammer A.M., Sonne C., Letcher R.J., Born E.W., Dietz R. (2009). Is dietary mercury of neurotoxicological concern to wild polar bears (*Ursus maritimus*)?. Environ. Toxicol. Chem..

[30] Haines K.J.R., Evans R.D., O’Brien M., Evans H.E. (2010). Accumulation of mercury and selenium in the brain of river otters (*Lontra canadensis*) and wild mink (*Mustela vison*) from Nova Scotia, Canada. Sci. Total Environ..

[31] Krey A., Kwan M., Chan H.M. (2012). Mercury speciation in brain tissue of polar bears (*Ursus maritimus*) from the Canadian Arctic. Environ. Res..

[32] Krey A., Kwan M., Chan H.M. (2014). In vivo and in vitro changes in neurochemical parameters related to mercury concentrations from specific brain regions of polar bears (*Ursus maritimus*). Environ. Toxicol. Chem..

[33] Krey A., Ostertag S.K., Chan H.M. (2015). Assessment of neurotoxic effects of mercury in beluga whales (*Delphinapterus leucas*), ringed seals (*Pusa hispida*), and polar bears (*Ursus maritimus*) from the Canadian Arctic. Sci. Total Environ..

[34] Scheuhammer A., Braune B., Chan H.M., Frouin H., Krey A., Letcher R., Loseto L., Noël M., Ostertag S., Ross P. (2015). Recent progress on our understanding of the biological effects of mercury in fish and wildlife in the Canadian Arctic. Sci. Total Environ..

[35] Rodrigues S., Coelho J.P., Bandeira V., Barros T., Duarte A.C., Fonseca C., Pereira M.E. (2014). Mercury bioaccumulation in the egyptian mongoose (*Herpestes ichneumon*): Geographical, tissue, gender and age differences. Water Air Soil Pollut..

[36] Komov V.T., Ivanova E.S., Gremyachikh V.A., Poddubnaya N.Y. (2016). Mercury content in organs and tissues of indigenous (*Vulpes vulpes* L.) and invasive (*Nyctereutes procyonoides* Gray.) species of canids from areas near Cherepovets (North-Western Industrial Region, Russia). Bull. Environ. Contam. Toxicol..

[37] Lanocha N., Kalisinska E., Kosik-Bogacka D.I., Budis H., Podlasinska J., Jedrzejewska E. (2014). Mercury levels in raccoons (*Procyon lotor*) from the Warta Mouth National Park, Northwestern Poland. Biol. Trace Elem. Res..

[38] Kalisinska E., Lanocha-Arendarczyk N., Kosik-Bogacka D., Budis H., Podlasinska J., Popiolek M., Pirog A., Jedrzejewska E. (2016). Brains of native and alien mesocarnivores in biomonitoring of toxic metals in Europe. PLoS ONE.

[39] Rodríguez-Jorquera I.A., Vitale N., Garner L., Perez-Venegas D.J., Galbán-Malagón C.J., Duque-Wilckens N., Toor G.S. (2017). Contamination of the upper class: Occurrence and effects of chemical pollutants in terrestrial top predators. Curr. Pollut. Rep..

[40] Lazarus M., Sekovanić A., Orct T., Reljić S., Kusak J., Jurasović J., Huber Đ. (2017). Apex predatory mammals as bioindicator species in environmental monitoring of elements in Dinaric Alps (Croatia). Environ. Sci. Pollut. Res..

[41] Lazarus M., Orct T., Sergiel A., Vranković L., Marijić V.F., Rašić D., Reljić S., Aladrović J., Zwijacz-Kozica T., Zięba F. (2020). Metal(loid) exposure assessment and biomarker responses in captive and free-ranging European brown bear (*Ursus arctos*). Environ. Res..

[42] Takeuchi H., Taki Y., Nouchi R., Yokoyama R., Kotozaki Y., Nakagawa S., Sekiguchi A., Iizuka K., Hanawa S., Araki T. (2021). Lead exposure is associated with functional and microstructural changes in the healthy human brain. Commun. Biol..

[43] Lazarus M., Sekovanić A., Orct T., Reljić S., Jurasović J., Huber Đ. (2018). Sexual maturity and life stage influences toxic metal accumulation in Croatian brown bears. Arch. Environ. Contam. Toxicol..

[44] Bechshoft T., Derocher A.E., Viengkone M., Routti H., Aars J., Letcher R.J., Dietz R., Sonne C., Jenssen B.M., Richardson E. (2018). On the integration of ecological and physiological variables in polar bear toxicology research: A systematic review. Environ. Rev..

[45] Malvandi H., Ghasempouri S.M., Esmaili-Sari A., Bahramifar N. (2010). Evaluation of the suitability of application of golden jackal (*Canis aureus*) hair as a noninvasive technique for determination of body burden mercury. Ecotoxicology.

[46] Cattet M.R.L., Caulkett N.A., Obbard M.E., Stenhouse G.B. (2002). A body-condition index for *Ursids*. Can. J. Zool..

[47] Lazarus M., Sekovanić A., Reljić S., Kusak J., Kovačić J., Orct T., Jurasović J., Huber D. (2014). Selenium in brown bears (*Ursus arctos*) from Croatia: Relation to cadmium and mercury. J. Environ. Sci. Health Part A Toxic/Hazardous Subst. Environ. Eng..

[48] Romanić S.H., Klinčić D., Kljakovic-Gašpić Z., Kusak J., Reljić S., Huber D. (2015). Organochlorine pesticides and polychlorinated biphenyl congeners in wild terrestrial mammals from Croatia: Interspecies comparison of residue levels and compositions. Chemosphere.

[49] Rodríguez-Estival J., Mateo R. (2019). Exposure to anthropogenic chemicals in wild carnivores: A silent conservation threat demanding long-term surveillance. Curr. Opin. Environ. Sci. Health.

[50] Chapron G., Kaczensky P., Linnell J.D.C., von Arx M., Huber D., Andrén H., López-Bao J.V., Adamec M., Álvares F., Anders O. (2014). Recovery of large carnivores in Europe’s modern human-dominated landscapes. Science.

[51] Trouwborst A., Krofel M., Linnell J.D.C. (2015). Legal implications of range expansions in a terrestrial carnivore: The case of the golden jackal (*Canis aureus*) in Europe. Biodivers. Conserv..

[52] Sienkiewicz T., Sergiel A., Huber D., Maślak R., Wrzosek M., Podgórski P., Reljić S., Paśko Ł. (2019). The brain anatomy of the brown bear (Carnivora, *Ursus arctos* L., 1758) Compared to that of other carnivorans: A cross-sectional study using MRI. Front. Neuroanat..

[53] Huber D., Kusak J., Majić-Skrbinšek A., Majnarić D., Sindičić M. (2008). A multidimensional approach to managing the European brown bear in Croatia. Ursus.

[54] Stoneberg R.P., Jonkel C.J. (1966). Age determination of black bears by cementum layers. J. Wildl. Manag..

[55] Gipson P.S., Ballard W.B., Nowak R.M., Mech L.D. (2000). Accuracy and precision of estimating age of gray wolves by tooth wear. J. Wildl. Manag..

[56] Marti I., Ryser-Degiorgis M.P. (2018). A tooth wear scoring scheme for age estimation of the Eurasian lynx (*Lynx lynx*) under field conditions. Eur. J. Wildl. Res..

[57] Knott E.J., Bunnefeld N., Huber D., Reljić S., Kereži V., Milner-Gulland E.J. (2014). The potential impacts of changes in bear hunting policy for hunting organisations in Croatia. Eur. J. Wildl. Res..

[58] Wikenros C., Gicquel M., Zimmermann B., Flagstad Ø., Åkesson M. (2021). Age at first reproduction in wolves: Different patterns of density dependence for females and males. Proc. R. Soc. B Biol. Sci..

[59] Mech L. (1970). The Wolf: The Ecology and Behavior of an Endangered Species.

[60] Lazarus M.V., Sekovanić A., Kljaković-Gašpic Z., Orct T., Jurasović J., Kusak J., Reljić S., Huber D. (2013). Cadmium and lead in grey wolf liver samples: Optimisation of a microwaveassisted digestion method. Arh. Hig. Rada Toksikol..

[61] Krebs N., Langkammer C., Goessler W., Ropele S., Fazekas F., Yen K., Scheurer E. (2014). Assessment of trace elements in human brain using inductively coupled plasma mass spectrometry. J. Trace Elem. Med. Biol..

[62] Huber Đ., Manen F.T., Vonk J., Shackelford T. (2019). Bear Morphology. Encyclopedia of Animal Cognition and Behavior.

[63] Puls R. (1994). Mineral Levels in Animal Health. Diagnostic Data.

[64] Monclús L., Shore R.F., Krone O. (2020). Lead contamination in raptors in Europe: A systematic review and meta-analysis. Sci. Total Environ..

[65] Ma W., Beyer W.N., Meador J.P. (2011). Lead in mammals. Environmental Contaminants in Biota: Interpreting Tissue Concentrations.

[66] Souza M.J., Ramsay E.C., Donnell R.L. (2013). Metal accumulation and health effects in raccoons (*Procyon lotor*) associated with coal fly ash exposure. Arch. Environ. Contam. Toxicol..

[67] Beyer W.N., Connor E.E., Gerould S. (1994). Estimates of soil ingestion by wildlife. J. Wildl. Manag..

[68] García M.H.D.M., Hernández Moreno D., Soler Rodríguez F., Beceiro A.L., Álvarez L.E.F., López M.P. (2011). Sex- and age-dependent accumulation of heavy metals (Cd, Pb and Zn) in liver, kidney and muscle of roe deer (*Capreolus capreolus*) from NW Spain. J. Environ. Sci. Health Part A Toxic/Hazardous Subst. Environ. Eng..

[69] Nordberg M., Nordberg G.F. (2022). Metallothionein and cadmium toxicology—Historical review and commentary. Biomolecules.

[70] Nordberg G., Nogawa K., Nordberg M. (2015). Cadmium. Handbook on the Toxicology of Metals.

[71] Wang B., Du Y. (2013). Cadmium and its neurotoxic effects. Oxid. Med. Cell. Longev..

[72] Malavolta M., Cipriano C., Costarelli L., Giacconi R., Tesei S., Muti E., Piacenza F., Pierpaoli S., Larbi A., Pawelec G. (2008). Metallothionein downregulation in very old age: A phenomenon associated with cellular senescence?. Rejuvenation Res..

[73] Bridges C.C., Zalups R.K. (2005). Molecular and ionic mimicry and the transport of toxic metals. Toxicol. Appl. Pharmacol..

[74] Yu H.-T., Zhen J., Leng J.Y., Cai L., Ji H.L., Keller B.B. (2021). Zinc as a countermeasure for cadmium toxicity. Acta Pharmacol. Sin..

[75] Huber D., Kulier I., Poljak A., Devčić-Kuhar B. (1993). Food intake and mass gain of hand-reared brown bear cubs. Zoo Biol..

[76] Hissa R. (1997). Physiology of the European brown bear (*Ursus Arctos Arctos*). Ann. Zool. Fennici.

[77] Vahter M., Åkesson A., Lidén C., Ceccatelli S., Berglund M. (2007). Gender differences in the disposition and toxicity of metals. Environ. Res..

[78] Llop S., Lopez-Espinosa M.J., Rebagliato M., Ballester F. (2013). Gender differences in the neurotoxicity of metals in children. Toxicology.

[79] Zaidi Z.F. (2010). Gender differences in human brain: A review. Open Anat. J..

[80] Tyler C.R., Allan A.M. (2014). The effects of arsenic exposure on neurological and cognitive dysfunction in human and rodent studies: A review. Curr. Environ. Health Rep..

[81] Reglero M.M., Taggart M.A., Monsalve-González L., Mateo R. (2009). Heavy metal exposure in large game from a lead mining area: Effects on oxidative stress and fatty acid composition in liver. Environ. Pollut..

[82] Berzas Nevado J.J., Rodríguez Martin-Doimeadios R.C., Mateo R., Rodríguez Fariñas N., Rodríguez-Estival J., Patiño Ropero M.J. (2012). Mercury exposure and mechanism of response in large game using the almadén mercury mining area (Spain) as a case study. Environ. Res..

[83] Vighi M., Borrell A., Aguilar A. (2017). Bone as a surrogate tissue to monitor metals in Baleen whales. Chemosphere.

[84] Peraza M.A., Ayala-Fierro F., Barber D.S., Casarez E., Rael L.T. (1998). Effects of micronutrients on metal toxicity. Environ. Health Perspect..

[85] Hudson L.G., Cooper K.L., Atlas S.R., King B.S., Jian L.K., States C. (2015). Arsenic interaction with zinc finger motifs. Arsenic: Exposure Sources, Health Risks, and Mechanisms of Toxicity.

[86] Porcella D.B., Zillioux E.J., Grieb T.M., Newman J.R., West G.B. (2004). Retrospective study of mercury in raccoons (*Procyon lotor*) in South Florida. Ecotoxicology.

[87] Woshner V.M., O’Hara T.M., Bratton G.R., Suydam R.S., Beasley V.R. (2001). Concentrations and interactions of selected essential and non-essential elements in bowhead and Beluga whales of Arctic Alaska. J. Wildl. Dis..

[88] Hoekstra P.F., Braune B.M., Elkin B., Armstrong F.A.J., Muir D.C.G. (2003). Concentrations of selected essential and non-essential elements in Arctic fox (*Alopex lagopus*) and wolverines (*Gulo gulo*) from the Canadian Arctic. Sci. Total Environ..

[89] Rush S.A., Borgå K., Dietz R., Born E.W., Sonne C., Evans T., Muir D.C.G., Letcher R.J., Norstrom R.J., Fisk A.T. (2008). Geographic distribution of selected elements in the livers of polar bears from Greenland, Canada and the United States. Environ. Pollut..

[90] Dietz R., Riget F., Born E.W. (2000). An assessment of selenium to mercury in Greenland marine animals. Sci. Total Environ..

[91] Khan M.A.K., Wang F. (2009). Mercury-selenium compounds and their toxicological significance: Toward a molecular understanding of the mercury-selenium antagonism. Environ. Toxicol. Chem..

[92] Toscano C.D., Guilarte T.R. (2005). Lead neurotoxicity: From exposure to molecular effects. Brain Res. Rev..

[93] Słota M., Wąsik M., Stołtny T., Machoń-Grecka A., Kasperczyk S. (2022). Effects of environmental and occupational lead toxicity and its association with iron metabolism. Toxicol. Appl. Pharmacol..

