# Supplementary material for: Lead and Other Trace Element Levels in Brains of Croatian Large Terrestrial Carnivores: Influence of Biological and Ecological Factors
Source: Toxics. 2022 Dec 20;11(1):4. doi: 10.3390/toxics11010004 (PMC9865836; doi:10.3390/toxics11010004)
Supplement: Supplementary file 1 [file toxics-11-00004-s001.zip › toxics-2028435-supplementary.pdf]

Supplementary Material:

Lead and Other Trace Element Levels in Brains of Croatian Large Terrestrial Carnivores: Influence of Biological and Ecological Factors

Table S1. Recent literature data for selected elements (mean, median) in brain of large and medium-sized terrestrial mammalian carnivores and humans expressed on a dry mass basis[1].

Species	Country/region	Sampling year	Sampled brain part	N	As (μg/kg)	Ca (mg/kg)	Cd (μg/kg)	Cu (mg/kg)	Fe (mg/kg)	Hg (μg/kg)	Pb (μg/kg)	Se (μg/kg)	Zn (mg/kg)	Reference
Brown bear *Ursus arctos*	Croatia	2015-2018	brain stem	114	8.10, 5.62	286, 245	16.2, 12.6	10.2, 9.57	93.5, 78.4	9.62, 7.93	93.2, 41.2	424, 413	30.4, 29.2	this study
Polar bear *Ursus maritimus*	East Grenland, Ittoqqortoormiit/ Scoresby Sound area	1999-2001	brain stem	82						340[mean]		710[mean]		Basu et al. 2009
Polar bear *Ursus maritimus*	East Grenland, Ittoqqortoormiit	2008	averaged for 11 brain regions	107						600[mean]				Desforges et al. 2021
Polar bear *Ursus maritimus*	Nunavik, Canada	2000-2003	cerebellum	24						230, 230				Krey et al. 2012
			frontal lobe	24						280, 290				Krey et al. 2012
			brain stem	24						120, 120				Krey et al. 2012
Grey wolf *Canis lupus*	Croatia	2016-2017	brain stem	8	5.27, 4.70	560, 460	3.22, 1.98	6.94, 7.27	148, 125	9.41, 7.53	29.5, 28.4	486, 491	28.9, 27.9	this study
Golden jackal *Canis aureus**	Croatia	2017	brain stem	2	6.47, 97.6	332, 502	0.664, 7.34	12.2, 16.8	56.0, 123	6.38, 273	10.9, 64.0	517, 608	32.1, 59.8	this study
Eurasian lynx *Lynx lynx*	Croatia	1998-2022	brain stem	3	9.96, 6.79	640, 670	2.19, 2.01	12.5, 12.2	83.8, 64.6	37.9, 24.4	9.59, 11.9	542, 591	43.1, 40.2	this study
Badger *Meles meles*	NW Poland	2009-2013	cerebrum	6			35[med]			124[med]	305[med]			Kalisinska et al. 2016
Red fox *Vulpes vulpes*	NW Russia, Vologda district	2007-2011	not specified	6						111				Komov et al. 2016
Red fox *Vulpes vulpes*	NW Poland	2009-2014	cerebrum	14			8[med]			23[med]	277[med]			Kalisinska et al. 2016
Raccoon *Procyon lotor*	NW Poland	2010-2013	cerebrum	29/17			26[med]			145[med]	470[med]			Kalisinska et al. 2016
Raccoon *Procyon lotor*	NW Poland	2009-2011	not specified	13						140, 80				Lanocha et al. 2014
Raccoon *Procyon lotor***	East Tennessee, USA	2009-2010	not specified	30		187-227		13.3-14.4	65.9-66.6	31.4-61.0		925-1165	51.6-60.9	Souza et al. 2013
Raccoon dog *Nyctereutes procyonoides*	NW Russia, Vologda district	2007-2011	not specified	14						111				Komov et al. 2016
Raccoon dog *Nyctereutes procyonoides*	NW Poland	2010-2013	cerebrum	12/15			36[med]			150[med]	184[med]			Kalisinska et al. 2016
Egyptian mongoose *Herpestes ichneumon*	Portugal	2011-2012	not specified	29						325[mean]				Rodrigues et al. 2014
Human *Homo sapiens*	Austria		averaged for 13 brain regions	11		338[mean]		21.4[mean]	298[mean]				44.0[mean]	Krebs et al. 2014
Human *Homo sapiens*	India		averaged for 12 brain regions	8	3530[mean]	603[mean]	101[mean]	17.4[mean]	139[mean]		2712[mean]		39.2[mean]	Rajan et al. 1997
Human *Homo sapiens* ***	World		various brain regions		7-6550		90-642	7.39-103	98-962	62-1147	330-3730	370-1200	27-132	Ramos et al. 2021

[1]levels recalculated on a dry mass took into account 73% H_2O (Basu et al. 2009; Krey et al. 2012)

**raw data for two individuals were indicated

***range of median values across three locations

****range of mean values reported for 14 brain regions in various studies across the globe

Table S2. Results of linear regression modelling of associations of trace elements in brown bear brain with sex, age group, body condition index (BCI) and season[1].

Dependent	Independent	N	b (95% CI)	p	adjusted R^2
As (log)	(Intercept)	110	0.586 (0.446, 0.726)	<0.001	0.005
	Sex				
	Male		-0.131 (-0.261, -0.001)	0.049	
	Female		0		
	Age group		NS		
	BCI		NS		
	Season		NS		
Ca (log)	(Intercept)	110	4.29 (4.20, 4.37)	<0.001	-0.03
	Sex		NS		
	Age group		NS		
	BCI		NS		
	Season		NS		
Cd (log)	(Intercept)	110	1.14 (0.996, 1.29)	<0.001	0.200
	Sex		NS		
	Age group				
	Cubs&Yearlings		-0.488 (-0.712, -0.264)	<0.001	
	Subadults		NS		
	Adults		0		
	BCI		NS		
	Season		NS		
Cu (log)	(Intercept)	110	0.911 (0.818, 1.00)	<0.001	0.107
	Sex		NS		
	Age group				
	Cubs&Yearlings		NS		
	Subadults		0.164 (0.051, 0.276)	0.005	
	Adults		0		
	BCI		NS		
	Season		NS		
Fe (log)	(Intercept)	110	3.11 (3.02, 3.21)	<0.001	-0.029
	Sex		NS		
	Age group		NS		
	BCI		NS		
	Season		NS		
Hg (log)	(Intercept)	110	0.836 (0.708, 0.964)	<0.001	0.029
	Sex		NS		
	Age group		NS		
	BCI		NS		
	Season		NS		
Se (log)	(Intercept)	110	4.72 (4.66, 4.78)	<0.001	0.020
	Sex		NS		
	Age group		NS		
	BCI		NS		
	Season		NS		

Zn (log)	(Intercept)	110	2.09 (2.04, 2.15)	<0.001	0.080
	Sex		NS		
	Age group				
	Cubs&Yearlings		0.104 (0.022, 0.186)	0.014	
	Subadults		NS		
	Adults		0		
	BCI		NS		
	Season		NS		

[1]Sex was coded as 1 for males and 2 for females; age group was coded as 1 for cubs&yearlings, 2 for subadults and 3 for adults; season was coded as 1 for spring and 2 for fall.
